# A scientific future shared with AI

**DOI:** 10.1371/journal.pbio.3003274

**Published:** 2025-06-30

**Authors:** Renee Hoch, Joanna Clarke

**Affiliations:** 1 Public Library of Science, San Francisco, California, United States of America and CambridgeUnited Kingdom

## Abstract

AI tools now exist to aid almost every aspect of the research process, from hypothesis generation and data analysis to manuscript drafting and publication. This Editorial examines what the future might hold for researchers and publishers as AI use continues to increase.

## Teaser

Artificial intelligence (AI) tools now exist to aid almost every aspect of the research process, from hypothesis generation and data analysis to manuscript drafting and publication. What does the future hold for researchers and publishers as AI use continues to increase?

Since Chat GPT’s launch in 2022, artificial intelligence (AI) has dominated conference programs, debates and discussions as the research and publishing communities have explored the opportunities and challenges introduced by large language model generative AI (genAI) tools. Within a short timeframe, genAI tools have become widely used by researchers to support activities such as drafting text, translation, language editing and knowledge discovery.

While genAI has brought the topic to the forefront of public discussions, AI itself is not new. Non-generative AI tools have been widely used for years and have proven valuable assets in research, enabling discoveries through complex analyses of large datasets and the generation and testing of hypotheses beyond what humans can themselves achieve [1,2]. We are running toward a future in which AI is likely to be embedded as a standard part of the research process, yet the technology is outpacing the development of guardrails, regulation and training [3,4]. While the research community experiments with incorporating AI into their work, many have begun to question how AI will change the future of science and science communication. Where will humans fit into the era of AI-assisted research? What are the rules? And do we need to redefine authorship?

In this issue of *PLOS Biology*, Nina Miolane [5] and Tatsuya Amano and colleagues [6] look at potential roles for AI in research and science publishing, respectively. The two areas of use demand different considerations, but in both phases AI needs to be viewed as an assistive tool that requires human oversight, not as a replacement for the human researcher. Nina Miolane describes AI as, “…the most overachieving and wildly enthusiastic intern; one who works at superhuman speed, never sleeps, and eagerly devours mountains of data. They hold exceptional potential, but without proper guidance anchored in scientific knowledge, they are more likely to set the lab on fire than to push science forward” [5]. Human researchers are needed to provide that guidance and to critically evaluate and contextualize new ideas, develop and test hypotheses and evaluate results, all the while applying their field-specific expertise, creativity and knowledge of ethical norms and policies.

Once data are generated, AI can help expedite the research communication process so that researchers spend less time drafting material that could be written by a chat bot and have more time to plan, conduct and analyze experiments. However, it is important to bear in mind that there are aspects of research communication for which genAI use is not appropriate: for example, the interpretation and discussion of results and their implications for the field should reflect a researcher’s own ideas. Moreover, it is critical that researchers are aware of, and abide by, journal policies and industry standards for ethical and responsible genAI use. Our recent interactions with researchers have indicated that there are notable knowledge gaps in these areas.

**Fig 1:**
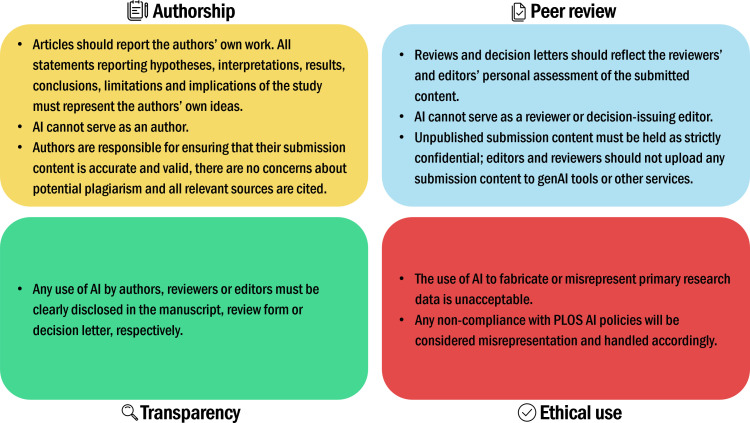
Key elements of PLOS’s policies on AI use. For more information see PLOS’s full policies on AI Tools and Technologies, AI tools in Peer Review and Authorship.

The current standards for AI use in scholarly communications reflect position statements issued by the Committee on Publication Ethics and World Association of Medical Editors in 2023 [7,8]. They did not forbid AI use, but they noted that AI tools should not be listed as authors and called for transparent disclosure, ethical use and author accountability. Many journals and publisher policies followed suit (see Fig 1 for a summary of PLOS’s policies on AI use). More detailed guidance on ethical and responsible AI use was issued in 2023 by the International Association of Scientific, Technical and Medical Publishers [9] and in 2025 by Wiley [10]. Raising researchers’ awareness of these resources and of evolving AI policies is an ongoing challenge for the industry.

An important aspect of AI literacy and training is understanding the risks as well as the benefits. Using genAI to draft content for research proposals, grant applications and manuscripts can introduce unreliable content, and may compromise the security and confidentiality of intellectual property and sensitive data. Before using a genAI tool it is important to review the tool’s Terms and Conditions: if they do not provide assurance that uploaded content will not be ingested into the tool’s dataset or otherwise utilized by the tool in the future, then using that tool for writing or translation may expose the unpublished original research (including any sensitive content) to reuse by others.

Authors are personally accountable for their submissions, including any content prepared with AI support. When using genAI, the onus is on authors to critically evaluate genAI outputs and edit as needed to ensure that their final product is reliable, well-founded, well-referenced and an accurate representation of their research and ideas. This due diligence may require a substantial time investment, but it is crucial to ensure the reliability and integrity of their work and adherence to journal policies. GenAI outputs reflect training data from varied source types, which may include peer-reviewed published literature as well as blogs, news articles, unvetted preprints and even retracted work. If sources are not provided with the AI tool’s outputs it may be difficult to verify the content, add references, avoid plagiarism and detect/remove ‘hallucinations’ (false, erroneous or irrelevant statements and references that are sometimes incorporated into genAI outputs).

Looking beyond individual-level contributions, a major benefit that AI provides is in helping to address global disparities in research and research communication. Many researchers around the world regularly have to write, read or evaluate work outside of their primary languages. As discussed by Amano and colleagues [6], AI translation and language tools have the potential to level the playing field for non-native English speakers by enabling them to write and review manuscripts in their own language. Furthermore, research findings and ideas published in other languages can be difficult to locate and are often missing from scholarly discussions that take place in English [11]. This issue could potentially be addressed by genAI tools; however, the outputs of such tools will only be as inclusive as their inputs, and the articles included in genAI training sets can be a major source of systemic bias. Moreover, researchers around the globe do not have equal access to AI tools and the computing power needed to support them. These issues may be addressed in the future through advances in AI and global efforts to improve access to AI in resource-limited settings, but in the meantime AI may only serve to widen disparities rooted in resource availability.

As useful as AI tools are, they can be problematic in the wrong hands. The same tools that can facilitate the writing process for legitimate researchers can be used by bad actors to rapidly fabricate manuscripts and peer reviews. It is likely not a coincidence that in the genAI era publishers are seeing an increase in large-scale publication ethics issues, including peer review integrity rings, authorship integrity issues and paper mills [12,13]. These fraudulent practices offer researchers a means of securing rapid, yet problematic, publications, and may ensnare unknowing authors by advertising as submission or peer review support services.

In this context, genAI presents a major risk to the integrity of the scholarly record, and undisclosed genAI use can lead readers, editors and reviewers to question the reliability, integrity and provenance of articles and peer review reports. This in turn can trigger integrity investigations at the journal or institutional level, and may prompt interventions that delay or impede the publication of legitimate research. By contrast, transparent disclosure of AI use, as is required by many journals’ AI policies (including *PLOS Biology*’s), can establish and foster trust, both in individual contributions and in AI-supported communications more broadly. Some have expressed concerns about disclosure requirements, worried about potential reviewer bias against AI-supported submissions; authors are encouraged to contact journals with concerns about specific decisions. We anticipate that this issue will dissipate as AI use disclosures become more common, AI policies become increasingly aligned across journals and researchers become more familiar with applicable policies and standards.

Like it or not, AI is here to stay. It will continue to improve and become embedded in research and publishing as more applications emerge, bringing many benefits that accelerate research and knowledge dissemination. As we move forward in the era of AI-supported science and take advantage of these new opportunities, we need to remember the limitations and potential risks of AI and stay apprised of updates to the tools themselves and to applicable policies, regulations and best practice standards.
